# Childhood Bullying Victimization, Substance Use and Criminal Activity among Adolescents: A Multilevel Growth Model Study

**DOI:** 10.3390/ijerph20010770

**Published:** 2022-12-31

**Authors:** Jungup Lee, Mijin Choi, Margaret M. Holland, Melissa Radey, Stephen J. Tripodi

**Affiliations:** 1 Department of Social Work, National University of Singapore, Singapore 117570, Singapore; 2 School of Social Work, Texas State University, San Marcos, TX 78666, USA; 3 Division of Social Work, University of Wyoming, Laramie, WY 82071, USA; 4 College of Social Work, Florida State University, Tallahassee, FL 32306, USA

**Keywords:** childhood bullying victimization, substance use, criminal activity, adolescence, young adulthood

## Abstract

Background: This study aims to examine the effects of childhood bullying victimization (CBV) on substance use and criminal activity among adolescents over time. In addition, it identifies the moderating effects of gender and race/ethnicity on the associations of CBV with substance abuse and criminal activity in adolescence and young adulthood. Methods: This study included 8984 adolescents aged 12 to 18 years (Mage = 14.22 years) assessed biennially at four time points utilizing the National Longitudinal Survey of Youth 1997. The two-level hierarchical linear modeling was employed to test the effects of CBV on substance use and criminal activity. Results: The incidence of substance use increased over time throughout adolescence to young adulthood, while that of criminal activity decreased. CBV increased the risks of cigarette use, marijuana use, and criminal activity. Gender and race/ethnicity significantly moderated the effect of CBV on alcohol use and alcohol binges. The effect of CBV on alcohol use was stronger among females than males. Among Hispanic adolescents, CBV was more strongly related to alcohol use and binges compared to non-Hispanic White. Conclusion: Findings suggest the need for early intervention for children at high risk of being bullied to reduce later substance abuse and involvement in criminal activities. Considering the moderating effects of gender and ethnicity on the associations, target-specified intervention and prevention programs are also required. Further studies focusing on the lifelong effects of CBV beyond adolescence are recommended.

## 1. Introduction

Bullying, defined as a persistent aggressive behavior intimidating others [1], is a salient problem confronting a large proportion of children and adolescents in the United States. It is estimated that about 20% of students are bullied during their time in school [2]. Many studies focusing on the association between bullying behaviors and negative life outcomes have evaluated the psychological impacts of bullying, such as behavioral problems [3], mental health issues [4,5,6], and suicidal ideation [7,8,9]. Bullying victimization has also been reported to be associated with adolescents’ social delinquency: substance abuse and involvement in criminal activities [10,11,12,13].

### 1.1. Coping with Bullying

Adolescents who are bullied in school experience various types of negative emotional conditions [14] and may seek convenient coping strategies to “feel better” [15]. For this reason, many studies have demonstrated that bullying victimization increases the risk of substance use [12,13,16]. One study, for instance, indicated that victims of bullying were at an elevated risk of alcohol and drug use, which were utilized as negative coping strategies [17]. Another study reported that cyberbullying victimization was positively associated with cigarette and alcohol use among sixth- to tenth-grade students [13].

Bullying victimization may also result in aggressive or criminal behaviors [18], which can be another type of coping strategy to deal with negative emotions [19]. Barboza [10] examined adolescents’ (ages 12–18) engagement in school delinquency and aggressive behaviors according to the existence and types of bullying victimization they had experienced. The findings showed that victims of severe bullying reported the highest likelihood of school delinquencies (e.g., avoiding school activities, skipping classes, and carrying a gun to school). In addition, victims of face-to-face bullying were at the highest risk for involvement in physical fights compared to non-victims and victims of cyberbullying [10].

### 1.2. Connecting Negative Coping Strategies to Theory

Understanding the nature of adolescents’ delinquency is crucial and requires additional consideration of their developmental process. Moffitt’s [20] developmental theory uses a dual taxonomy to classify adolescents’ antisocial behavior into “life-course-persistent” and “adolescent-limited” behaviors. People with life-course-persistent behaviors (childhood-onset) exhibit problematic behaviors from early childhood, which persist continuously and steadily throughout their entire life course. In contrast, people with adolescent-limited behaviors (adolescent-onset) exhibit prosocial behaviors in childhood, problematic behaviors in adolescence resulting from age-normative experiences, and prosocial behaviors in adulthood. This adolescent-limited antisocial pathway likely stems from a maturity gap when adolescents desire to fulfill adult roles and engage in adult privileges but are not yet allowed to fulfill their desires. This gap may result in problematic behaviors only relevant in adolescence. In addition, adolescent-limited crime is more likely to be influenced by peers compared to life-course-persistent crime. This theory can be applied to explain that adolescent problematic behavior may either result from (1) life-course-persistent behaviors that may be exacerbated by CBV or (2) adolescent-limited behaviors caused by CBV, which would resolve in adulthood. Therefore, it is imperative to investigate the unique consequences of CBV on adolescent behavioral problems, as well as to explore the long-term effects of CBV on problematic behaviors into adulthood.

### 1.3. Mixed Evidence between CBV and Criminal Activity and Substance Use

Despite this need for further research to investigate the effects of CBV on problematic behaviors into adulthood, most CBV studies focused on the association with a risk of criminal activity and substance use in later life are based on cross-sectional data with a few notable exceptions [21,22,23,24]. For instance, Gorman et al. [25] evaluated students’ (ages 13–16) CBV experiences, as well as long-term outcomes on mental health and labor market outcomes of those same people when they turned 25 years old. Using propensity score matching to compare people who had and people who had not been bullied, the findings indicated that people who had been bullied in adolescence had worse mental health, more unemployment, and less income than people who had not been bullied in adolescence [25]. Relating these findings to the current study, CBV-caused criminal activity and substance use may be a mechanism to explain the worse mental health and labor market outcomes.

In addition, Eriksen et al. [22] implemented a longitudinal study where parents of children were surveyed at 14 weeks gestation and twice when their children were between nine and 13 years old and found that CBV led to decreased GPA and that more severe bullying led to even worse GPA. This finding may reveal a mechanism by which CBV, through lowered GPA, causes later criminal activity and substance use. Another study that analyzed longitudinal cohort data collected at three time points (kindergarten, third grade, and ninth grade) reported that CBV marginally predicted later substance use [21]. Wolke et al. [24] documented that CBV, which was assessed for adolescents between the ages of 9 to 16, was significantly associated with tobacco use and felony charges in later young adulthood for bully victims (children who were both bullies and bullied). However, they did not find a relationship between CBV and risky and illegal behaviors for children who were only victims. Similarly, another study analyzing longitudinal data that followed fourth-grade boys until age 34 reported that CBV (at ages 10–12) resulted in higher odds of suicide attempts, arrests, clinically significant mental health symptoms, and tobacco use in young adulthood (at ages 20–32) for bully victims, compared to youths who were not the subjects of bullying [23]. These findings give support for the theory that children use bullying as a negative coping strategy to handle their negative emotions—in these cases, related to being victimized by bullying [14,15]. This nuance in understanding CBV may be a possible mechanism that leads to increased risk of criminal activity and substance misuse.

### 1.4. Relationship between CBV, Behavioral Problems, and Gender and Race/Ethnicity

Previous studies have shown that gender and race/ethnicity are related to bullying victimization and behavioral problems [16,26,27,28,29]. Luk et al. [16] reported that the association of bullying victimization with substance abuse was contingent upon the level of depression only for female students. Connolly’s study [30] using the National Longitudinal Survey of Youth 1997 (NLSY97) found that male victims of bullying were more likely than non-bullied males to use/smoke cigarette and marijuana, whereas female victims were more prevalent in cigarette use than their non-bullied females from adolescence through young adulthood. Another study conducted by Glassner and Cho [31] also examined the gender differences in the association between CBV and substance use using the NLSY found that CBV escalated the risk of substance use in adolescence and young adulthood only for males but not females. Moreover, several studies have found that African American elementary students were less likely to be victimized compared to their counterparts [1,26,28]. Further research is needed that assesses the long-term effects of CBV on substance use and criminal activity over time while evaluating, the moderating effects of gender and race/ethnicity. 

## 2. Current Study

The current study aims to examine the effects of CBV on multiple forms of substance use (cigarette use, alcohol use, alcohol binges, and marijuana use) and criminal activity among adolescents over time. Additionally, the study investigates how the association of CBV with substance use and criminal activity in adolescence and young adulthood differs by gender and racial/ethnic group. Research hypotheses are: (1) the probability of multiple forms of substance use will differ over time from adolescence to young adulthood; (2) the probability of involvement in criminal activity will differ over time; (3) CBV will be positively related to multiple forms of substance use over time; (4) CBV will be positively related to involvement in criminal activity over time; (5) the effects of CBV on substance use and criminal activity will be contingent on gender; and (6) the effects of CBV on substance use and criminal activity will be contingent on race/ethnicity. 

## 3. Method

### 3.1. Data and Sampling

This study used archival data from the National Longitudinal Survey of Youth 1997 (NLSY97), a national representative survey of U.S. youth from 12 to 18 years of age. Sponsored by the U.S. Bureau of Labor Statistic (BLS), the NLSY has been conducted annually through youth and parent interviews in English or Spanish by professional field interviewers using Computer-Assisted Personal Interviews (CAPI) and Audio Computer-Assisted Self Interviewing (ACASI) for sensitive topics, such as substance use and criminal activity. The NLSY has provided extensive information on respondents’ labor market behaviors, educational experiences, delinquency, and family backgrounds. The retention rate in the NLSY study is high, with 79.5% at the seventh round. The current study was reviewed and approved by the university’s Institutional Review Board. This study utilized longitudinal data from Waves 1, 3, 5, and 7 of the NLSY (four different time points; 1997, 1999, 2001, and 2003) as these waves selected for the study analysis provided relevant items for investigating adolescents’ criminal activity and the effects of CBV on substance use and criminal activity. The final sample included 8984 adolescents aged 12–18 at Time 1 to examine the patterns of substance use and criminal activity across four time points (Time 1 through Time 4) and the long-term effects of CBV on substance use and criminal activity over time.

### 3.2. Measures

#### 3.2.1. Dependent Variables of Interests

We measured five dependent variables: (1) four substance use outcomes, which include cigarette use, alcohol use, alcohol binges, and marijuana use, and (2) criminal activity. For the four variables of substance use, the respondents were asked to rate how many days they have ever experienced cigarette use, alcohol use, alcohol binges (consuming five or more drinks on an occasion), and marijuana use in the past 30 days (ranging from 0 to 30). Criminal activity was measured by six items asking the respondents if they had ever committed the following behaviors over the past year: intentional destruction of property, theft of items worth less than $50, theft of items worth more than $50, other property crimes, attacking someone with an intent to do serious harm, and selling illegal drugs. These six criminal activity items were dichotomously coded as ‘no’ (0) versus ‘yes’ (1) and summed, with total occurring scores ranging from zero to six (α = 0.78). All five outcomes were characterized as count data at each time. 

#### 3.2.2. Childhood Repeated Bullying Victimization (CBV)

CBV was measured by a dichotomous indicator (0 = no and 1 = yes), asking the respondents if they had ever been the victim of repeated bullying before age 12 (at Time 1). 

#### 3.2.3. Time-Fixed Covariates

Time-fixed covariates were selected for factors that might influence the effect of CBV on subsequent substance use and criminal activity. These included delinquent peers in childhood, mother’s educational level in childhood, and behavioral and emotional problems. Delinquent peers in childhood was measured with five items, asking the respondents to rate what percentage of their peers in school smoked cigarettes, got drunk at least once a month, belonged to a gang, used illegal drugs, and cut classes or skipped school at Time 1. Response options ranged from 0 = almost none (less than 10%), 1 = about 25%, 2 = about half (50%), 3 = about 75%, to 4 = almost all (more than 90%). All items were summed and characterized as a continuous variable. Mother’s educational level in childhood was categorized into three response options: 0 = less than high school, 1 = high school/GED, and 2 = college or above. Behavioral and emotional problems were measured with four items adapted from the Child Behavior Checklist (CBCL) [32]. Separate scales of the behavioral and emotional problems were administered to male and female adolescents [33,34]. Male participants were asked to rate their degree of four problematic behaviors (“you have trouble concentrating or paying attention”, “you don’t get along with other kids”, “you lie or cheat”, and “you are unhappy, sad, or depressed”). Female participants were asked to rate their degree of difficulty with two of the same behaviors (“you lie or cheat” and “you are unhappy, sad, or depressed”) and two different behaviors (“your school work is poor” and “you have trouble sleeping”). Both scales rated on a 3-point scale (0 = not true, 1 = somewhat/sometimes true, and 2 = often true) and conducted a total score that ranged from 0 to 8. Further, two demographics, gender (0 = male, 1 = female) and race/ethnicity (0 = non-Hispanic White, 1 = non-Hispanic Black, 2 = Hispanic/Latino) were used as moderators. Other categories of race/ethnicity were excluded due to low presentation. 

#### 3.2.4. Time-Varying Covariates

We measured the following five time-varying covariates at each wave: (1) gangs in neighborhood (0 = no, 1 = yes); (2) siblings or friends in gangs (0 = no, 1 = yes); (3) living area (0 = rural area, 1 = urban area); (4) living with biological parents (0 = living with others, 1 = living with both biological parents); (5) household size (the number of family members; range 1–17); and (6) age (in years). 

### 3.3. Analytic Strategy 

Hierarchical linear modeling (HLM) was used for the longitudinal data analyses [35]. As missing data were treated as Missing at Random, full information maximum likelihood (FIML) was used to handle missing data. The four different time points across Wave 1 through 7 were considered to be clustered within each adolescent, and the analyses consisted of two-level hierarchical structures including within adolescents and between adolescents. Substance use and criminal activity as the outcomes were measured as count data with many zero and skewed distributions with a variance. Thus, a multilevel growth model with an over-dispersed Poisson sampling distribution was used for this study [36]. In the Poisson models, the average rates of multiple forms of substance use and criminal activity are determined by exponentiating the intercept terms, as shown in the event rate ratios (ERRs).

## 4. Results

### 4.1. Descriptive Characteristics 

Table 1 presents the descriptive statistics for the key variables across the four time points. At Time 1, both male and female students were equally distributed. Nearly 52% were non-Hispanic White, 27% were non-Hispanic Black, and 21% were Hispanic. Additionally, 3 in 4 students lived in urban areas and 51% lived with biological parents. The average age of the respondents was 14.22 (SD = 1.46, range 12–18) years and the average household size was 4.56 (SD = 1.52, range 1–17). Among the respondents, nearly 20% experienced bullying victimization as a child. Almost 46% indicated there were gangs in their neighborhood at Time 1, but the prevalence rates decreased over time to 13% at Time 4. Similarly, the prevalence of having siblings or friends in gangs declined over time from 21% at Time 1 to 5% at Time 4. As shown in Table 2, male students were more prevalent in substance use and criminal activity than their female counterparts at the four time points, except for cigarette use, which was only significant at Time 3 and Time 4. Meanwhile, non-Hispanic White students reported the highest prevalence of substance use at Times 2–4 and criminal activity at Time 2 compared to other racial/ethnic students. 

### 4.2. Substance Use and Criminal Activity in Multiple Growth Models

#### 4.2.1. Model 1: Unconditional Growth Models

To test the first and second hypotheses, unconditional growth models were conducted with a time predictor (Model 1). Model construction began with a linear growth model that included only a time indicator at level 1 to test the linear growth of substance use and criminal activity outcomes and significant variation in the outcomes over time. Model 1 was described as:

Level-1 Model: E(*Y_ti_*|*π_i_*) = λ*_ti_*

log[λ*_ti_*] = η*_ti_*

η*_ti_* = *π*_0*i*_ + *π_1i_*×(*TIME_ti_*)

Level-2 Model: *π*_0*i*_ = *β*_00_ + *r*_0*i*_

*π*_1*i*_ = *β*_10_ + *r*_1*i*_

Mixed Model: η*_ti_* = *β*_00_ + *β*_10_×*TIME_ti_* + *r*_0*i*_ + *r*_1*i*_×*TIME_ti_*

where *π*_0*i*_ refers to the initial probability of outcomes (i.e., cigarette use, alcohol use, alcohol binges, marijuana use, and criminal activity) for individual *i* at Time 1, *π*_1*i*_ refers to the growth rate from Time 1 through Time 4, *β*_00_ represents the average initial status, *β*_10_ represents the average rate of change (or the average slope of *TIME* across adolescents), *r*_0*i*_ represents the deviation of initial status, and *r*_1*i*_ refers to the deviation of rate of change. *TIME* was centered around the grand mean. Level-1 variance is 1/λ*_ti_.*

As shown in Table 3, unconditional growth models (Model 1) were performed to characterize the trajectories of multiple forms of substance use and criminal activity over the four time points. The results indicated that the ERRs of four types of substance use (i.e., cigarette use, alcohol use, alcohol binges, and marijuana use) increased over time (ERRs = 1.74, 1.92, 1.77, and 1.34; *p* < 0.001, respectively), whereas the ERR of criminal activity decreased over time (ERR = 0.50, *p* < 0.001). More specifically, the slope of the incidents of substance use escalated remarkably over time, increasing by 74% for cigarette use, 92% for alcohol use, 77% for alcohol binges, and 34% for marijuana use per 2-year increase in age. Conversely, the slope of the incidence of criminal activity declined by 50% per 2-year interval. 

#### 4.2.2. Model 2: Conditional Growth Models at Level 1 (Within-Individual Effects)

To test the third and fourth hypotheses, conditional growth models were conducted with time-fixed and time-varying covariates at level 1 (Model 2). Model 2 was described as:

Level-1 Model: η*_ti_* = *π*_0*i*_ + *π*_1*i*_×(*TIME_ti_*) + *π*_2*i*_×(*CBV_i_*) + *π*_3*i*_×(*DP_i_*) + *π*_4*i*_×(*MEDU_i_*) +

*π*_5*i*_×(*BP_ti_*) + *π*_6*i*_×(*HH_ti_*) + *π*_7*i*_×(*LIVIN_ti_*) + *π*_8*i*_×(*GNEI_ti_*) + *π*_9*i*_×(*SIBLING_ti_*) + *π*_10*i*_×(*AGE_ti_*) + *π*_11*i*_×(*BEP_i_*)

Level-2 Model: *π*_0*i*_ = *β*_00_ + *r*_0*i*_

*π*_1*i*_ = *β*_10_ + *r*_1*i*_

*π*_2*i*_ = *β*_20_ + *r*_2*i*_

*π*_3*i*_ = *β*_30_, *π*_4*i*_ = *β*_40_,…, *π*_11*i*_ = *β*_110_

Mixed Model: η*_ti_* = *β*_00_ + *β*_10_×*TIME_ti_* + *β*_20_×*CBV_i_* + *β*_30_×*DP_i_* + *β*_40_×*MEDU_i_* +

*β*_50_×*BP_ti_* + *β*_60_×*HH_ti_* + *β*_70_×*LIVIN_ti_* + *β*_80_×*GNEI_ti_* + *β*_90_×*SIBLING_ti_* + *β*_100_×*AGE_ti_* + *β*_110_×*BEP_i_* + *r*_0*i*_ + *r*_1*i*_×*TIME_ti_* + *r*_2*i*_×*CBV_i_*

where *TIME*, *CBV*, *DP*, *MEDU*, *BP*, *HH*, *LIVIN*, *GNEI*, *SIBLING, AGE, and BEP* were centered around the grand mean. Level-1 variance is 1/λ*_ti_.*

Table 4 presents the findings of the conditional growth models with time-fixed and time-varying covariates at level 1 (within-individual effects; Model 2). When controlling for all covariates, the time-trend for multiple forms of substance use revealed a positive rate ratio, whereas criminal activity showed a negative rate ratio, which was consistent as shown in Model 1. CBV increased the slope of the ERRs of cigarette use, marijuana use, and criminal activity after controlling for other variables (ERRs = 1.45, *p* < 0.001, 1.24, *p* < 0.01, and 1.50, *p* < 0.001, respectively). However, there was no significant impact of CBV on alcohol use and alcohol binges. 

Further, both childhood delinquent peer association and mother’s educational level at Time 1 affected the slope of the ERRs of all forms of substance use and criminal behavior. Adolescents who resided with both biological parents had fewer events of alcohol binges (ERR = 0.88, *p* < 0.01), and those in a larger household size had fewer events of alcohol use (ERR = 0.96, *p* < 0.05) and alcohol binges (ERR = 0.95, *p* < 0.05). Those with siblings or friends in gangs were at a higher risk of alcohol use (ERR = 1.16, *p* < 0.05), alcohol binges (ERR = 1.29, *p* < 0.01), and criminal activity (ERR = 1.80, *p* < 0.001). Interestingly, the presence of gangs in an adolescent’s neighborhood increased the risk of criminal activity (ERR = 1.41, *p* < 0.001) but reduced the risk of cigarette use (ERR = 0.78, *p* < 0.001) and alcohol use (ERR = 0.86, *p* < 0.05). Older adolescents were at an increasing risk of cigarette use (ERR = 1.07, *p* < 0.001), alcohol use (ERR = 1.20, *p* < 0.001), and alcohol binges (ERR = 1.19, *p* < 0.001) later in life but at a reduced risk of marijuana use (ERR = 0.97, *p* < 0.001) and criminal activity (ERR = 0.93, *p* < 0.001) compared to younger counterparts. In addition, adolescents with behavioral and emotional problems at Time 1 were at an increased risk of alcohol use (ERR = 1.09, *p* < 0.05), alcohol binges (ERR = 1.11, *p* < 0.05), marijuana use (ERR = 1.23, *p* < 0.01), and criminal activity (ERR = 1.31, *p* < 0.001) over time. 

#### 4.2.3. Overall Trends of Substance Use and Criminal Activity by Childhood Bullying Victimization

**Figure 1:**
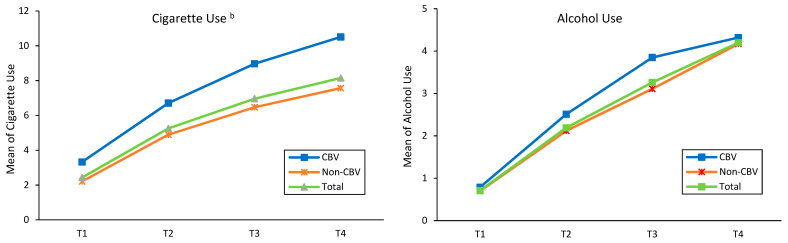

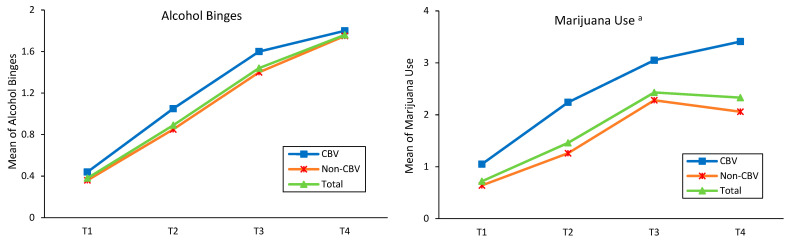
Trend in rates of substance use by CBV from Time 1 to Time 4. ^a^ *p* < 0.05, ^b^ *p* < 0.01.

**Figure 2:**
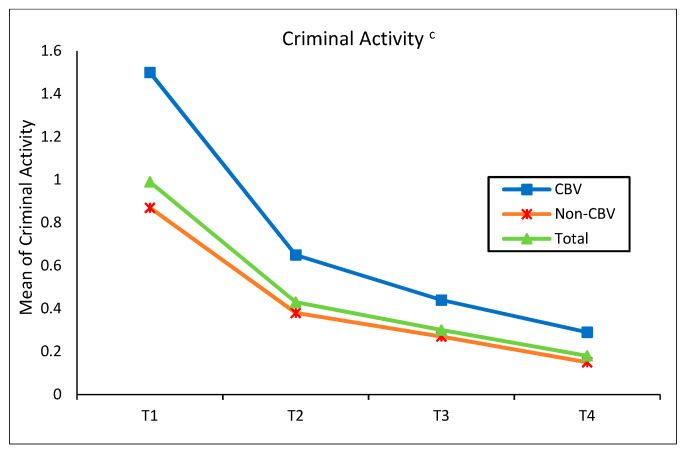
Trend in rates of criminal activity by CBV from Time 1 to Time 4. ^c^ *p* < 0.001.

Figure 1 depicts the prevalence of multiple forms of substance use for total sample, adolescents exposed to CBV, and adolescents not exposed from Times 1 to 4. The results indicated that all types of substance use among adolescents increased over time, except for marijuana use. The prevalence of marijuana use increased from Time 1 through Time 3 but slightly reduced at Time 4. Regarding the comparison of CBV and non-CBV, adolescents who experienced CBV reported higher rates of all types of substance use, compared to those who had non-CBV at the four time points. As shown in Figure 2, conversely, the prevalence of adolescents’ criminal activity gradually decreased over time. Adolescents who were bullied in childhood were more prevalent in criminal activity than those who were not bullied in childhood through the four time points. In particular, the rate of criminal activity for adolescents with CBV was almost two times higher than that for the non-bullied counterparts at Time 1.

To examine whether changes in substance use and criminal activity over time differ for two groups (CBV and non-CBV), the moderation analyses were performed with the two-way interaction term (time×CBV). Additionally, the gender differences in the moderating effects were investigated. Table 5 presents the changes of the parameter estimates in substance use and criminal activity over time by CBV for the total, male and female groups. The slope changes in cigarette use, marijuana use, and criminal activity over time differed significantly between bullied youths and non-bullied youths. Compared with non-bullied youths, bullied youths reported the faster slope changes in the levels of cigarette use (ERR = 1.46, *p* < 0.01) and marijuana use (ERR = 1.52, *p* < 0.05) and the level of criminal activity (ERR = 1.15, *p* < 0.01). The slope changes in the level of criminal activity between CBV and non-CBV over time were significant for both male and female groups. However, the slope changes in level of cigarette use by CBV over time were significant only for males but not females, while those in level of marijuana use by CBV over time were significant only for females but not males. 

#### 4.2.4. Model 3: Conditional Growth Models with Interacting Covariates at Level 2 (Between-Individual Effects)

To test the fifth and sixth hypotheses, conditional growth models were performed with interacting covariates (i.e., gender and race/ethnicity) at level 2 (Model 3). The level 1 model in Model 3 is identical to that in Model 2. The model equations for Model 3 were described as:

Level-1 Model: η*_ti_* = *π*_0*i*_ + *π*_1*i*_×*(TIME_ti_*) + *π*_2*i*_×(*CBV_i_*) + *π*_3*i*_×(*DP_i_*) + *π*_4*i*_×(*MEDU_i_*) +

*π*_5*i*_×(*BP_ti_*) + *π*_6*i*_×(*HH_ti_*) + *π*_7*i*_×(*LIVIN_ti_*) + *π*_8*i*_×(*GNEI_ti_*) + *π*_9*i*_×(*SIBLING_ti_*) + *π*_10*i*_×(*AGE_ti_*) + *π*_11*i*_×(*BEP_i_*)

Level-2 Model: *π*_0*i*_ = *β*_00_ + *β*_01_×(*GENDER_i_*) + *β*_02_×(*ETHNIC_i_*) + *β*_03_×(*BLACK_i_*) + *r*_0*i*_

*π*_1*i*_ = *β*_10_ + *β*_11_×(*GENDER_i_*) + *β*_12_×(*ETHNIC_i_*) + *β*_13_×(*BLACK_i_*) + *r*_1*i*_

*π*_2*i*_ = *β*_20_ + *β*_21_×(*GENDER_i_*) + *β*_22_×(*ETHNIC_i_*) + *β*_23_×(*BLACK_i_*) + *r*_2*i*_

*π*_3*i*_ = *β*_30_, *π*_4*i*_ = *β*_40_,…, *π*_11*i*_ = *β*_110_

Mixed Model: η*_ti_* = *β*_00_ + *β*_01_×*GENDER_i_* + *β*_02_×*ETHNIC_i_* + *β*_03_×*BLACK_i_* + *β*_10_*×TIME_ti_* + *β*_11_×*GENDER_i_×TIME_ti_* + *β*_12_×*ETHNIC_i_×TIME_ti_* + *β*_13_×*BLACK_i_×TIME_ti_* + *β*_20_×*CBV_i_* +

*β*_21_×*GENDER_i_*×*CBV_i_* + *β*_22_×*ETHNIC_i_*×*CBV_i_* + *β*_23_×*BLACK_i_*×*CBV_i_* + *β*_30_×*DP_i_* +

*β*_40_×*MEDU_i_* + *β*_50_×*BP_ti_* + *β*_60_×*HH_ti_* + *β*_70_×*LIVIN_ti_* + *β*_80_×*GNEI_ti_* + *β*_90_×*SIBLING_ti_* + *β*_100_×*AGE_ti_* + *β*_110_×*BEP_i_* + *r*_0*i*_ + *r*_1*i*_×*TIME_ti_* + *r*_2*i*_×*CBV_i_*

where *TIME*, *CBV*, *DP*, *MEDU*, *BP*, *HH*, *LIVIN*, *GNEI*, *SIBLING, AGE, BEP, GENDER*, *ETHNIC*, and *BLACK* were centered around the grand mean. Level-1 variance is 1/λ*_ti_.*

Table 4 also shows the results of the conditional growth models with interacting covariates including gender and race/ethnicity at leve1 2 (between-individual effects; Model 3). Gender and race/ethnicity were significant covariates in influencing the slope of risk ratios of some substance use over Time 1 through Time 4. More specifically, the slope of the risk ratio of cigarette use for female adolescents was lower than that for male adolescents (ERR = 0.90, *p* < 0.05). Compared to non-Hispanic White counterparts, Hispanic adolescents reported the lower slope of the risk ratios of alcohol use (ERR = 0.94, *p* < 0.01) and alcohol binges (ERR = 0.89, *p* < 0.05) over time, while non-Hispanic Black adolescents had the higher slope of the risk ratios of cigarettes use (ERR = 1.18, *p* < 0.01) and criminal activities (ERR = 1.06, *p* < 0.05) over time. 

**Figure 3:**
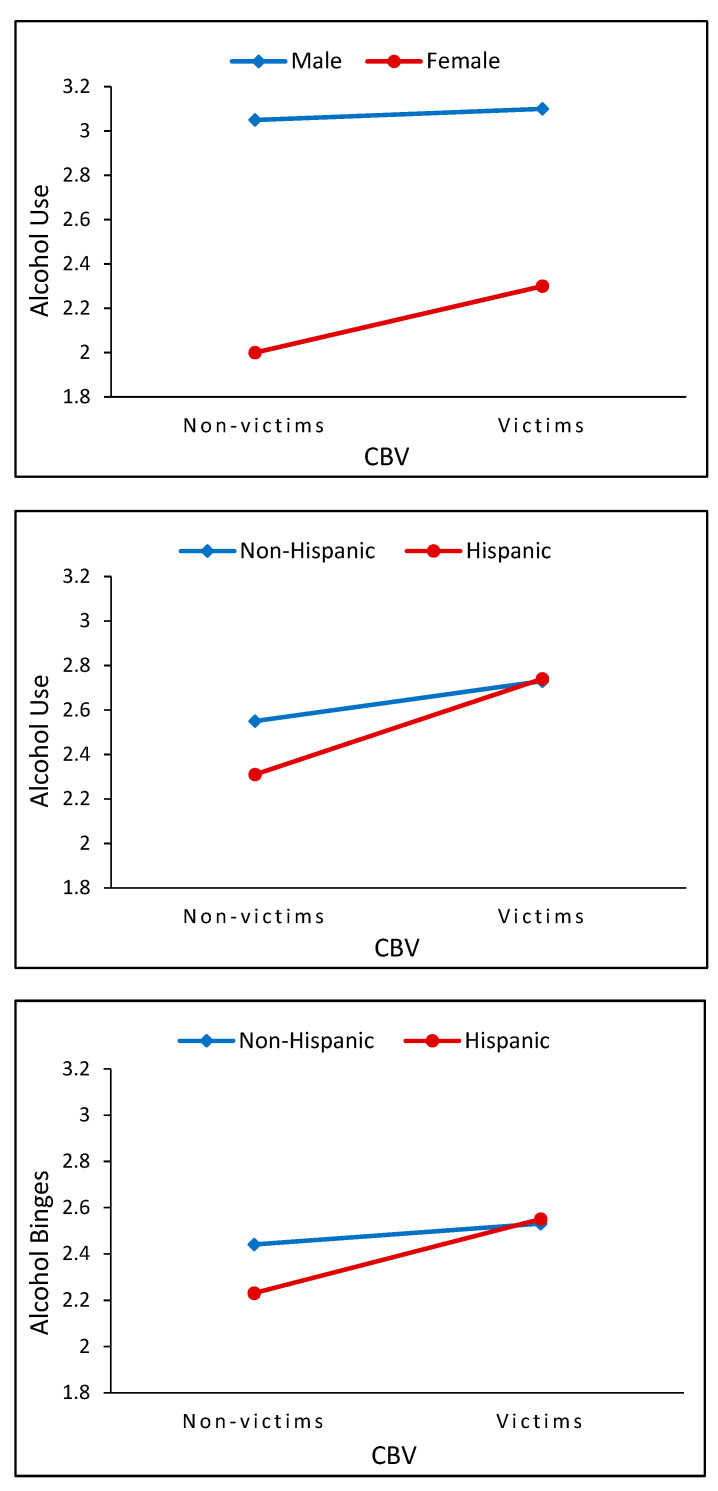
The moderating roles of gender on the effect of CBV on alcohol use (**top**; *p* < 0.05), ethnicity on the effect of CBV on alcohol use (**middle**; *p* < 0.01), and ethnicity on the effect of CBV on alcohol binges (**bottom**; *p* < 0.05).

As shown in Figure 3, gender and race/ethnicity significantly moderated the effects of CBV (gender×CBV and race/ethnicity×CBV) on alcohol use and alcohol binges in adolescence and young adulthood. The slope changes in the effect of CBV on alcohol use manifested more strongly among female adolescents than male adolescents (ERR = 1.22, *p* < 0.05). Compared to non-Hispanic White, Hispanic youth showed the larger slope changes in effects of CBV on alcohol use (ERR = 1.40, *p* < 0.01) and alcohol binges (ERR = 1.37, *p* < 0.05). 

## 5. Discussion

This study identifies how CBV influences multiple forms of substance use (cigarette use, alcohol use, alcohol binges, and marijuana use) and involvement in criminal activity from adolescence through young adulthood. By using a large longitudinal dataset of a nationally representative sample of U.S. adolescents, findings contribute to the literature and support developmental theory as a theoretical framework. Like other forms of adverse childhood experiences (ACEs), CBV has long-lasting effects on behaviors and health outcomes over time. 

This study revealed several findings. First, youth substance use demonstrated a positive linear growth rate over time. The linear growth of youth substance use is consistent with previous studies claiming that substance use increases in adolescence due to the likelihood of risk-taking traits [17,37,38]. However, the onset of alcohol use itself in adolescence cannot be underestimated. Studies have documented that early exposure to substance use is related to the increased risk of lifelong substance abuse disorder [39,40] that can interfere with adolescents’ neurodevelopment. Therefore, identification and prevention of risk factors that make adolescents vulnerable to substance abuse, such as early pubertal development (early maturation), impulsivity, lower parental education, and other preexisting conditions are necessary [40,41,42,43]. Childhood adversity is one of the key risk factors as well. Although Connell et al. [21] found that CBV has only minor effects on the onset of youth substance use, the present study shows that CBV is an underlying predictor of increased cigarette use, marijuana use, and criminal activity among adolescents over time. This may be consistent with previous studies reporting that the victims of bullying in childhood are at higher risk of smoking and drug use in adulthood than their non-victim counterparts [24]. Given the limited research on ongoing effects of childhood bullying on lifelong behavioral outcomes, further research on the later outcomes of childhood bullying over time is required. In addition, the identification of various risk factors for substance abuse and additional attention toward vulnerable adolescents who are exposed to risk factors are critical to reduce further development of behavioral problems. 

Next, the current study supports the previous study that indicated that adolescent criminal activity decreases over time [37]. Although there has been debate on delinquency trajectories throughout adolescence [44], this decline may be explained by the changing level of delinquency over life stages. In addition, the present study sample showed low involvement in criminal activity (M = 0.99 out of 6) at the average age of 14. However, considering that early onset of delinquency in adolescence is related to longer and more serious externalization of delinquency [33] and it leads to young adult crime [45], intervention for young adolescents to prevent their age-related normative delinquency from developing into later criminal activities is crucial. 

Third, the relationship between CBV and alcohol abuse increased more among female adolescents than male adolescents. Using the same dataset, a prior study also reported that male adolescents who were repeatedly bullied showed slower decline in marijuana use from adolescence to adulthood, suggesting that this sex difference could be associated with gendered coping strategies in response to stress: females are more likely to focus on support from other people who experience similar difficulties, while males are more likely to withdraw from relationships that act as sources of the stress [30]. Although this former study examined a different period of age ranges from the present study, it indicates that gender plays a vital role in interpreting the effects of CBV on substance use. 

In addition, the present study reveals that the effects of CBV on both alcohol use and alcohol binges are stronger among Hispanics than non-Hispanic Whites, but Hispanic adolescents appear to show less involvement in alcohol-related behaviors. In detail, the negative association of household size with alcohol use and binges shown in Model 2 disappeared after entering race/ethnicity (i.e., Hispanic) in Model 3. Considering how Hispanic families tend to have larger household sizes than other racial/ethnic families (t = 22.82, *p* < 0.001), family characteristics may be related to adolescents’ behaviors. Similarly, findings reported that the presence of siblings in gangs was related to increased probabilities of involvement in criminal activity. Petts [44] argues that family structure and process (e.g., parental affection and supervision) influence youth delinquency. The utilization of family characteristics that reflects cultural contexts could be implemented to design and provide effective programs. Overall, providing target-specified prevention and intervention programs considering adolescents’ victimized bullying experience, gender-specific traits (e.g., coping strategies), and the family environment is recommended. 

## 6. Limitations

Several limitations warrant closer scrutiny. First and foremost, the NLSY97 data utilized in this study was collected two decades ago (1997/98–2003/04). The emergence of technologies (e.g., prevalent use of smartphone and social media platforms) may have influenced youth behaviors, leading to new types of bullying and criminal involvement (e.g., cyberbullying). However, the findings of the current longitudinal study might be utilized as a fundamental framework for future studies to explore new types of youth risk behaviors and their consequences over time. A second limitation lies in attrition or exclusion bias, which is one of the key threats to internal validity, especially for longitudinal studies. For instance, adolescents who committed deviant behavior at Time 1 may tend to be at a higher risk of incarceration or probation, which can lead in turn to attrition from the data collection at Time 4. Of the total 8984 cases at Time 1, nearly 7142 cases retained through Time 4. Additionally, other crime measures (e.g., number of criminal activities, incarceration, recidivism) could be explained the rationale for the reduced trend of youth criminal activity. Future studies need to seek alternative strategies to decrease the attrition or exclusion rate in longitudinal research. 

A third limitation is that adolescents’ age range may lead to variations in their trajectories of substance use and criminal activity. For instance, the trajectory of substance use for adolescents aged from 12 years at Time 1 to 18 years at Time 4 may be different from those aged from 16 at Time 1 to 22 years at Time 4. Hence, it is cardinal to perform longitudinal studies in which the same cohort groups are followed over time in order to explicitly investigate changes in adolescents’ developmental trajectories and to consider the significant determinants of developmental trajectories in adolescents’ antisocial behavior [46]. Further longitudinal research with a cohort-sequential model is recommended for assessing more accurate variations of the developmental trajectories of substance use and criminal activity. A fourth limitation includes the self-reported survey data, which relied on youth’s perception and might increase the likelihood of social desirability bias, particularly as respondents were asked to report socially objectionable behaviors (e.g., CBV, criminal activity). Because most of the self-report measures in this study are sensitive in nature, a significant amount of overreporting or underreporting can occur. Future research should be considered to adopt the use of multiple informants (e.g., parent-report measures), yielding greater reliability. 

In addition, CBV was assessed using single-item indicators, which may have limited validity. Furthermore, Sullivan et al. [29] also reported that the relationships of peer victimization with externalizing aggression, alcohol use, and delinquency were differentiated according to the types of victimization and gender of students among the predominant sample of African American students. However, the current study used a single variable for representing CBV. Further research including more depth of information is recommended for validating the findings of the current study. Further, the research models in the current study does not account for any unobservable features. Future research should be highlighted with unobserved confounders, which might be driving the effect of CBV on substance use and criminal behavior. Finally, this study did not include possible predictors exhaustively: for instance, social support, which might play a protective role with regard to substance use and criminal activity. Further research with a more comprehensive frame is recommended. 

Despite the limitations above, this study contributes to useful knowledge on youth substance use and criminal activity. It examines trajectories of substance use and criminal activity among adolescents over time, considering other factors affecting behavioral problems, beyond risk-taking traits throughout adolescence. Findings could be applicable for youth health and social service research and practices, informing youth workers and school counsellors of the long-term detrimental effects of bullying behaviors on youths over time. Furthermore, these findings may provide insights for designing and implementing school-based bullying prevention and intervention programs for adolescents and young adults.

## Figures and Tables

**Table 1:** Descriptive statistics.

	Time 1	Time 2	Time 3	Time 4
Variables	M (*SD*)	M (*SD*)	M (*SD*)	M (*SD*)
Childhood bullying victimization (0–1)	0.20 (0.40)			
Delinquent peers in childhood (0–20)	8.48 (3.82)			
Mother educational level in childhood (0–4)	2.49 (1.41)			
Gangs in neighborhood (0–1)	0.46 (0.50)	0.44 (0.50)	0.17 (0.38)	0.13 (0.34)
Sibling or friends in gangs (0–1)	0.21 (0.41)	0.11 (0.32)	0.08 (0.27)	0.05 (0.23)
Age	14.22 (1.46)	16.86 (1.43)	18.92 (1.43)	20.88 (1.43)
Behavioral and emotional problems (0–8)	2.15 (1.58)			
Gender (female)	0.50 (0.50)			
Race/ethnicity				
Non-Hispanic White	0.52 (0.50)			
Non-Hispanic Black	0.27 (0.44)			
Hispanic	0.21 (0.40)			
Urban area (0–1)	0.75 (0.43)	0.74 (0.44)	0.77 (0.42)	0.79 (0.41)
Household size (1–17)	4.56 (1.52)	4.33 (1.61)	4.03 (1.72)	3.60 (1.73)
Biological parents (0–1)	0.51 (0.50)	0.47 (0.49)	0.40 (0.48)	0.28 (0.35)

Note. *N* = 8984 at Baseline (Time 1); *N* = 8466 at Time 2; *N* = 7879 at Time 3; and *N* = 7142 at Time 4.

**Table 2:** Descriptive statistics for substance use and criminal activity over time across gender and racial/ethnicity.

	Male	Female		White	Black	Hispanic	
	M (*SD*)	M (*SD*)	*t*	M (*SD*)	M (*SD*)	M (*SD*)	*F*
Cigarette use							
Time 1	2.47 (7.39)	2.37 (10.71)	0.54	3.50 (8.83)	1.00 (4.43)	1.50 (5.60)	79.10 ***
Time 2	5.42 (10.71)	5.31 (10.70)	0.44	7.42 (12.19)	2.77 (7.88)	3.57 (8.73)	133.22 ***
Time 3	7.49 (12.15)	6.49 (11.62)	3.41 **	9.20 (13.13)	4.40 (9.77)	4.66 (9.79)	127.31 ***
Time 4	8.69 (12.66)	7.15 (12.13)	4.99 ***	9.96 (13.42)	5.91 (11.06)	5.17 (10.25)	101.76 ***
Alcohol use							
Time 1	0.84 (2.78)	0.64 (2.34)	3.12 **	0.39 (1.58)	0.47 (2.25)	0.88 (2.85)	12.93 ***
Time 2	2.51 (5.01)	1.78 (3.85)	6.58 ***	2.72 (4.87)	1.22 (3.70)	1.97 (4.31)	64.25 ***
Time 3	3.90 (6.28)	2.54 (4.49)	10.03 ***	4.04 (5.91)	1.87 (4.32)	3.01 (5.57)	89.14 ***
Time 4	5.11 (6.93)	3.21 (5.08)	12.61 ***	5.17 (6.54)	2.51 (5.06)	3.77 (5.99)	110.36 ***
Alcohol binges							
Time 1	0.48 (2.12)	0.28 (0.52)	4.41 ***	0.17 (0.97)	0.21 (1.48)	0.46 (1.86)	9.75 ***
Time 2	1.23 (3.13)	0.64 (2.07)	8.96 ***	1.31 (3.14)	0.34 (1.50)	0.84 (2.57)	75.23 ***
Time 3	2.00 (4.35)	0.95 (2.61)	11.84 ***	1.99 (4.07)	0.59 (2.39)	1.35 (3.51)	86.80 ***
Time 4	2.39 (4.71)	1.06 (2.77)	13.86 ***	2.29 (4.38)	0.74 (2.61)	1.62 (3.89)	90.12 ***
Marijuana use							
Time 1	0.93 (4.26)	0.52 (2.98)	4.54 ***	0.26 (1.53)	0.66 (3.67)	0.64 (3.41)	1.63
Time 2	1.94 (6.26)	1.02 (4.16)	7.00 ***	1.78 (5.81)	1.01 (4.34)	1.36 (5.20)	12.10 ***
Time 3	3.12 (8.03)	1.58 (5.56)	9.02 ***	2.69 (7.29)	1.93 (6.49)	2.03 (6.60)	8.60 ***
Time 4	2.88 (7.76)	1.54 (5.65)	7.97 ***	2.55 (7.23)	1.98 (6.55)	1.59 (5.84)	10.66 ***
Criminal activity							
Time 1	1.27 (1.49)	0.71 (1.08)	17.39 ***	0.99 (1.31)	0.97 (1.28)	0.93 (1.33)	2.35
Time 2	0.54 (1.05)	0.31 (0.73)	10.35 ***	0.45 (0.95)	0.40 (0.87)	0.37 (0.24)	3.55 *
Time 3	0.37 (0.88)	0.20 (0.60)	9.44 ***	0.29 (0.78)	0.28 (0.71)	0.27 (0.76)	0.43
Time 4	0.25 (0.70)	0.11 (0.43)	9.62 ***	0.17 (0.57)	0.18 (0.57)	0.20 (0.66)	0.59

* p < 0.05, ** p < 0.01, *** p < 0.001.

**Table 3:** Unconditional growth models (Model 1).

	Cigarette Use	Alcohol Use	Alcohol Binges	Marijuana Use	Criminal Activity
	ERR (SE)	ERR (SE)	ERR (SE)	ERR (SE)	ERR (SE)
Fixed effect					
Intercept, *β*_00_	0.55 (0.04) ***	1.00 (0.02)	0.36 (0.03) ***	0.19 (0.04) ***	0.27 (0.02) ***
Slope, *β*_10_	1.74 (0.02) ***	1.92 (0.01) ***	1.77 (0.01) ***	1.34 (0.02) ***	0.50 (0.01) ***
Random effect (Variance)					
Intercept, *r_o_*	5.95 ***	1.79 ***	2.29 ***	4.21 ***	1.03 ***
Slope, *r*_1_	0.69 ***	0.30 ***	0.33 ***	0.63 ***	0.14
Model fit					
Deviance	103,673.82	93,306.26	69,528.11	131,111.08	54,301.79
χ^2^	36,418.46 ***	19,947.70 ***	8451.72 ***	2535.85 ***	4037.37 ***

Note. ERR = event rate ratio. *** p < 0.001.

**Table 4:** Conditional growth models with covariates at level 1 (Model 2) and with interacting covariates at level 2 (Model 3).

	Cigarette Use		Alcohol Use		Alcohol Binges		Marijuana Use		Criminal Activity	
	Model 2	Model 3	Model 2	Model 3	Model 2	Model 3	Model 2	Model 3	Model 2	Model 3
	ERR (SE)	ERR (SE)	ERR (SE)	ERR (SE)	ERR (SE)	ERR (SE)	ERR (SE)	ERR (SE)	ERR (SE)	ERR (SE)
Fixed effect										
Level-1										
Intercept, *β*_00_	0.58 (0.03) ***	0.57 (0.03) ***	1.01 (0.02)	1.00 (0.01)	0.37 (0.03) ***	0.34 (0.03) ***	0.18 (0.04) ***	0.19 (0.04) ***	0.25 (0.02) ***	0.24 (0.02) ***
Slope, *β*_10_	1.68 (0.02) ***	1.69 (0.02) ***	1.94 (0.02) ***	1.92 (0.02) ***	1.89 (0.02) ***	1.82 (0.02) ***	1.40 (0.03) ***	1.38 (0.03) ***	0.55 (0.02) ***	0.53 (0.02) ***
CBV, *β*_20_	1.45 (0.09) ***	1.42 (0.08) ***	1.01 (0.05)	1.01 (0.06)	1.02 (0.06)	1.01 (0.06)	1.24 (0.08) **	1.20 (0.08) **	1.50 (0.04) ***	1.47 (0.04) ***
DP, *β*_30_	1.15 (0.01) ***	1.18 (0.02) ***	1.12 (0.01) ***	1.15 (0.01) ***	1.05 (0.01) ***	1.07 (0.01) ***	1.18 (0.02) ***	1.20 (0.01) ***	1.09 (0.01) ***	1.11 (0.01) ***
MEDU, *β*_40_	1.06 (0.03) *	0.98 (0.03)	1.15 (0.01) ***	1.08 (0.02) ***	1.12 (0.02) ***	1.06 (0.02) **	1.15 (0.03) ***	1.11 (0.03) ***	1.08 (0.01) ***	1.04 (0.01) **
BP, *β*_50_	1.06 (0.03)	1.06 (0.03)	1.03 (0.02)	1.04 (0.02)	0.88 (0.03) **	0.89 (0.03) **	0.98 (0.05)	0.95 (0.05)	0.93 (0.02)	0.89 (0.02) *
HH, *β*_60_	0.97 (0.02)	0.98 (0.03)	0.96 (0.02) *	0.97 (0.02)	0.95 (0.02) *	0.97 (0.02)	1.01 (0.04)	1.02 (0.04)	1.00 (0.01)	1.01 (0.01)
LIVIN, *β*_70_	1.06 (0.08)	1.07 (0.08)	1.05 (0.06)	1.08 (0.06)	1.04 (0.07)	1.09 (0.07)	1.12 (0.15)	1.14 (0.15)	0.98 (0.04)	1.03 (0.03)
GNEI, *β*_80_	0.78 (0.05) ***	0.83 (0.05) ***	0.86 (0.06) *	0.89 (0.06)	0.97 (0.08)	1.00 (0.08)	0.94 (0.11)	0.90 (0.12)	1.41 (0.03) ***	1.43 (0.03) ***
SIBLING, *β*_90_	1.15 (0.09)	1.12 (0.09)	1.16 (0.07) *	1.10 (0.09)	1.29 (0.10) **	1.23 (0.11) *	1.25 (0.15)	1.25 (0.15)	1.80 (0.04) ***	1.81 (0.04) ***
Age, *β*_100_	1.07 (0.03) ***	1.04 (0.03) **	1.20 (0.04) ***	1.18 (0.05) ***	1.19 (0.07) ***	1.17 (0.07) ***	0.97 (0.04) ***	0.95 (0.05) ***	0.93 (0.08) ***	0.91 (0.07) ***
BEP, *β*_110_	1.09(0.08)	1.04 (0.07)	1.09 (0.03) *	1.12 (0.02) *	1.11 (0.03) *	1.12 (0.03) *	1.23 (0.04) **	1.23 (0.05) *	1.31 (0.02) ***	1.25 (0.02) ***
Level-2										
Female, *β*_11_		0.90 (0.03) *		0.95 (0.02)		0.95 (0.03)		1.01 (0.03)		0.98 (0.03)
Hispanic, *β*_12_		1.01 (0.05)		0.94 (0.03) **		0.89 (0.04) *		0.97 (0.05)		1.03 (0.03)
Black, *β*_13_		1.18 (0.05) **		0.97 (0.03)		0.93 (0.04)		1.02 (0.05)		1.06 (0.03) *
Female, *β*_21_		1.19 (0.15)		1.22 (0.10) *		1.20 (0.11)		1.27 (0.15)		1.11 (0.06)
Hispanic, *β*_22_		1.28 (0.21)		1.40 (0.13) **		1.37 (0.15) *		1.09 (0.21)		1.04 (0.09)
Black, *β*_23_		0.89 (0.18)		1.21 (0.11)		1.11 (0.13)		0.93 (0.16)		1.00 (0.06)
Random effect (Variance)										
Intercept, *r_o_*	5.24 ***	5.18 ***	1.70 ***	1.53 ***	2.24 ***	1.98 ***	4.24 ***	4.09 ***	0.85 ***	0.83 ***
Slope, *r*_1_	0.72 ***	0.63 ***	0.33 ***	0.32 ***	0.31 ***	0.33 ***	0.64 ***	0.65 ***	0.11	0.10
Model fit										
Deviance	106,201.43	102,898.32	93,021.43	92,587.98	69,768.98	68,225.84	96,641.87	96,985.93	53,640.90	53,345.07
χ^2^	497.56 ***	258.34 ***	412.96 ***	476.43 ***	366.54 ***	584.54 ***	458.35 ***	125.89 ***	1045.76 ***	408.89 ***

Note. Model 2 presents the conditional growth models at level 1 (within-individual effects); Model 3 presents the conditional growth models with interacting covariates at level 2 (be-tween-individual effects). ERR = event rate ratio; CBV = childhood bullying victimization; DP = delinquent peers in childhood; MEDU = mother educational level in childhood; BP = biological parents; HH = household size; LIVIN = living area; GNEI = gangs in neighborhood; SIBLING = sibling or friends in gangs; BEP = behavioral and emotional problems. * p < 0.05, ** p < 0.01, *** p < 0.001.

**Table 5:** Changes of the parameter estimates in substance use and criminal activity over time by CBV.

	Cigarette Use	Alcohol Use	Alcohol Binges	Marijuana Use	Criminal Activity
Time×CBV	ERR (SE)	ERR (SE)	ERR (SE)	ERR (SE)	ERR (SE)
Total	1.46 (0.13) **	0.84 (0.12)	0.93 (0.14)	1.52 (0.18) *	1.15 * (0.09) **
Male	1.64 (0.12) *	1.02 (0.14)	0.89 (0.12)	1.03 (0.17)	1.08 (0.11) ***
Female	1.26 (0.13)	0.80 (0.13)	0.95 (0.15)	1.67 (0.21) *	1.09 (0.10) ***

Note. ERR = event rate ratio. * p < 0.05, ** p < 0.01, *** p < 0.001.

## Data Availability

The NLSY97 data are available in U.S. Bureau of Labor Statistics, https://www.bls.gov/nls/getting-started/accessing-data.htm (accessed on 10 August 2021).
